# Intralesional Corticosteroid Administration in the Treatment of Keloids: A Scoping Review on Injection Methods

**DOI:** 10.1159/000529220

**Published:** 2023-01-19

**Authors:** Qi Yin, Juliette M.I. Louter, Frank B. Niessen, Susan Gibbs, Gizem Tasdemir-Kilic, Oren Lapid, Paul P.M. van Zuijlen, Albert Wolkerstorfer

**Affiliations:** ^a^Amsterdam UMC, Department of Dermatology, Amsterdam, The Netherlands; ^b^Amsterdam UMC, Department of Plastic, Reconstructive and Hand Surgery, Amsterdam, The Netherlands; ^c^Department of Molecular Cell Biology and Immunology, Amsterdam Institute for Infection and Immunity (AII), Amsterdam UMC, Vrije Universiteit, Amsterdam, The Netherlands; ^d^Department of Oral Cell Biology, Academic Centre for Dentistry Amsterdam (ACTA), University of Amsterdam and Vrije Universiteit, Amsterdam, The Netherlands; ^e^Burn Center and Department of Plastic, Reconstructive and Hand Surgery, Red Cross Hospital, Beverwijk, The Netherlands; ^f^Amsterdam UMC location University of Amsterdam, Pediatric Surgical Centre, Emma Children's Hospital, Amsterdam, The Netherlands; ^g^Amsterdam Movement Sciences (AMS) Institute, Amsterdam UMC, Amsterdam, The Netherlands

**Keywords:** Keloid, Corticosteroid, Triamcinolone, Intralesional corticosteroids, Injection

## Abstract

**Background:**

Intralesional corticosteroid administration (ICA) is a first-line treatment for keloids. However, its clinical results are still highly variable and often suboptimal. Treatment results may strongly be influenced by various operator-dependent factors. The aim of this study was to map the details of ICA in keloids described in randomized controlled trials (RCTs), hence presenting the scientific practice of a first-line treatment for keloids in the best available evidence.

**Summary:**

A systematic search was performed on PubMed, Ovid MEDLINE, Ovid EMBASE, and CENTRAL. Eligible studies were RCTs including patients with keloids treated with intralesional corticosteroids. Treatment and study design-related data were charted on a predefined form. Thirty-eight RCTs were included for data extraction. Triamcinolone acetonide was used in 37 (97.4%) studies. Dosing per cm<sup>2</sup> could only be compared among ten (26%) studies and varied from 1 to 20 mg. The maximum dose per session varied from 20 to 80 mg. Local anesthetics were administered in seven (20%) RCTs. Treatment intervals varied from weekly to monthly, with 4 weeks most frequently (50%) used. Needle size was reported in eleven (29%) studies and varied from 26 to 30-gauge. Syringe size was specified in four (11%) studies, being 1 mL. The injection level was described in eleven (29%) studies. Blanching as endpoint was reported in ten (26%) studies. Outcome measures varied widely, from height, surface area, or volume, to Vancouver Scar Scale, Patient and Observer Scar Assessment Scale, pain and itch scores, patient satisfaction, and different efficacy rates. Only six studies had a follow-up of ≥6 months. Recurrence was identified in two studies with 18 weeks and 1 year of follow-up. Adverse events were reported in 23 (61%) studies.

## Introduction

Keloids are fibroproliferative disorders resulting from chronic inflammation in the dermis and often cause pain, pruritus, a tight sensation, cosmetic concerns, and occasionally movement restriction [1, 2]. The quality of life is impacted, especially when pain, pruritus, or functional restrictions are experienced [3, 4]. Intralesional corticosteroid administration (ICA) is a first-line treatment in current practice, of which triamcinolone acetonide (TAC) is most commonly used [5]. Corticosteroids induce keloid regression through many different mechanisms, including but not limited to the inhibition of leukocyte and monocyte migration and phagocytosis resulting in reduced inflammation, increased vasoconstriction resulting in reduced oxygen and nutrient delivery to the wound bed, and the inhibition of keratinocytes and fibroblasts resulting in reduced re-epithelialization and new collagen formation [5]. Even though ICA is a first-line keloid treatment, clinical results of this treatment are still highly variable and often suboptimal [6, 7]. Treatment results may strongly be influenced by clinicians' preferences, such as the type, volume, and concentration of the corticosteroid, the number and interval of treatment sessions, the needle and syringe size, and the manual injection techniques. A search of PROSPERO, CENTRAL, and PubMed identified one systematic review from 2008 that aimed to include (randomized) controlled trials on the most effective concentration and treatment interval of intralesional TAC in keloids. However, the authors did not retrieve any studies that fit their inclusion and exclusion criteria [8]. To our knowledge, there is currently no consensus on different aspects of ICA in keloids among physicians. We hypothesized that a wide variety in this first-line treatment may exist in scientific practice as well. Identifying the (non)uniformity of intralesional corticosteroid treatment protocols is of an exploratory nature that is suited to a scoping review. The aim of this scoping review was to map the details of ICA in keloids described in randomized controlled trials (RCTs), hence presenting the scientific practice of a first-line treatment for keloids in the best available evidence up to date.

## Methods

This scoping review was developed in accordance with the methodological framework devised by the Joanna Briggs Institute [9]. Prior to starting this study, a predefined charting form was designed with items that we aimed to address. Registration of the review protocol in PROSPERO could not be performed as this is a scoping review. All other items of the Preferred Reporting Items for Systematic reviews and Meta-analyses extension for Scoping Reviews (PRISMA-ScR) checklist were addressed (online suppl. File 1; for all online suppl. material, see www.karger.com/doi/10.1159/000529220).

### Search Strategy

A systematic search was performed on PubMed, Ovid MEDLINE, Ovid EMBASE, and CENTRAL for articles from inception to January 17, 2022. The search combined terms on (1) keloid and (2) intralesional therapy or injection in title or abstract, and involved relevant MeSH terms and synonyms. Inclusion of terms on generic and chemical names of corticosteroids was not attempted, so that no corticosteroid treatment could be missed. In the context of a scoping review, no explicit outcomes were stated in the search, aiming to capture all relevant papers, irrespective of used outcome measures. The search was limited to studies written in English, French, Dutch, and German. The full electronic search is described in online supplementary 2.

### Inclusion and Exclusion Criteria

Following the search, all identified citations were imported into EndNote version 20 bibliographic management software. Two reviewers performed the title and abstract screening (Q.Y. and G.K.), and two reviewers (Q.Y. and G.K. or J.L.) independently assessed the full texts on eligibility. Discrepancies were resolved by a fourth reviewer (AW). Reference lists of the included articles were checked for additional studies of interest. Studies were eligible if they included (1) humans of any race, gender, and age, (2) with one or more keloids of any size, in any anatomic location, with any duration and with any etiology, and (3) were RCTs that involved intralesional corticosteroids. Only RCTs were included, because of the high level of evidence inherent to its study design. Articles on both keloids and hypertrophic scars were included only if separated data for keloids were available.

### Data Extraction

Data charting was performed by QY and GK in duplicate. Data collected on the study population included the used (1) diagnostic criteria for the keloid, (2) number of patients and keloids that received intralesional corticosteroids, (3) location of the keloid, and (4) country of research origin. Data collected on the treatment were details of the used (5) drugs and dosing, including the type, volume, and concentration of the corticosteroid, the number and interval of treatment sessions, and local anesthetics, (6) equipment, including the needle and syringe size, and (7) manual injection techniques including the level of injection, angle of injection, speed of injection, number of injections per session, and endpoint of infiltration. Additionally, study design-related data were collected, which included (8) outcome measures, (9) follow-up period and recurrence, and (10) adverse events. A concise summary was provided for each charted item.

### Critical Appraisal of Methodological Quality

A risk-of-bias assessment was performed by two reviewers (Q.Y. or J.L.) to determine the quality of current RCTs on this first-line treatment of keloids. Version 2 of the Cochrane risk-of-bias tool for randomized trials was used.

## Results

**Fig. 1:**
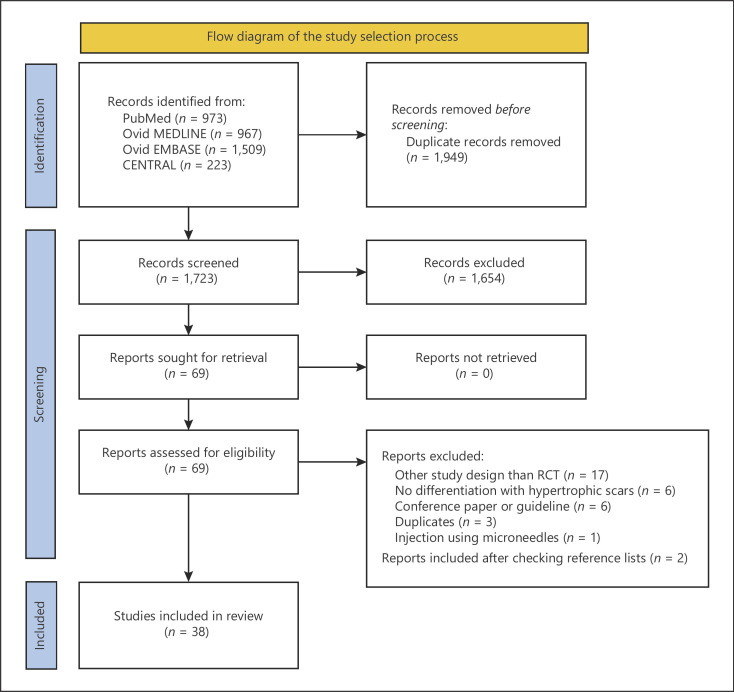
Flow diagram of the study selection process.

The search yielded 1,723 unique articles, of which 38 articles were included for data extraction (shown in Fig. 1 flowchart). Of these included studies, only one study compared the effect of two different doses of TAC directly [10], thirty-four studies investigated different therapies for keloids, with the TAC group being either the treatment or the control arm, and three studies primarily investigated the effect of local anesthetics on injection pain.

### Diagnostic Criteria

The diagnostic criteria were not described by twenty-four (63%) studies. Six studies (16%) separately presented data of both keloids and hypertrophic scars [11, 12, 13, 14, 15, 16], of which only three studies specified the criteria for distinction between them, with the keloid being an entity that extends over the margins of the original injury and the hypertrophic scar one that remains within the confines of the original injury [12, 14, 16]. Six studies on both keloids and hypertrophic scars were excluded, because data of two entities were pooled.

### Drugs and Dosing

TAC was used in 37 (97.4%) studies. Only one study used a combination of betamethasone disodium phosphate 2 mg/mL and betamethasone dipropionate 5 mg/mL [17]. The dose of TAC per cm^2^ of keloid could only be compared among ten (26%) studies and differed by a factor of 20 [6, 7, 10, 12, 17, 18, 19, 20, 21, 22] (Table 1). Whereas one study reported to inject 0.1 mL of TAC 10 mg/mL (dose: 1 mg TAC) per cm^2^ of keloid [12], other studies reported to inject 0.5 mL of TAC 40 mg/mL (dose: 20 mg TAC) per cm^2^ of keloid [6, 18, 19]. The used concentrations of TAC were 40 mg/mL (39%), 20 mg/mL (18%), 10 mg/mL (24%), and 5 mg/mL (2.7%). In five (13%) studies, the used concentration of TAC was unclear. The injected volume was only reported in twenty (53%) studies. The maximum dose administered per session was mentioned in twelve (32%) studies and varied from 20 to 80 mg (Table 1).

The total number of treatment sessions varied from one [20] to eight [11, 13, 16], with the median being four sessions. Reported treatment intervals differed from weekly [16, 23] to monthly [15, 17, 24, 25, 26, 27]. The most commonly described treatment interval was every 4 weeks (50%), followed by every 3 weeks (29%), weekly (15%), and every 2 weeks (6%). Six studies mentioned that treatment would be stopped if complete lesion flattening or clearance occurred [10, 18, 28, 29, 30, 31] (Table 1).

The effect of local anesthetics on injection pain was investigated by three studies [15, 21, 22] (Table 1). One study demonstrated that 1:1 mixture of lidocaine 1% and TAC did not alleviate needle puncture pain, whereas EMLA cream did [21]. Another study revealed that the mean VAS score of needle puncture and steroid infiltration was significantly lower among patients that received prior cooling with icepack, compared to no pre-treatment (*p* < 0.001) and lidocaine and prilocaine 25/25 mg/g (EMLA) cream (*p* < 0.05) [22]. The third study demonstrated that application of −10⁰C using a cooling device to the skin before injection leads to lower pain scores [15]. Among the RCTs that did not primarily focus on injection pain, local anesthetics were used in seven (20%) studies and included lidocaine 1% [11, 13], lidocaine 2% [19], and EMLA cream [32]. Two studies used a lidocaine-TAC mixture as treatment [32, 33]. Oral analgesics were administrated in one study [18].

### Equipment

Reported needle sizes were 26 (brown, 0.45 mm), 27 (medium gray, 0.40 mm), and 30-gauge (yellow, 0.30 mm), which were used in, respectively, one [17], seven [11, 12, 14, 16, 18, 19, 34], and three studies [28, 31, 35]. Twenty-seven (71%) studies did not report the needle size. The syringe size was only specified in four studies, being 1 mL [11, 16, 35, 36]. Seven (18%) studies reported the use of an insulin syringe, without mentioning its size [6, 18, 20, 23, 28, 31, 37] (Table 1).

### Manual Injection Techniques

The level of injection was only specified in eleven studies [11, 13, 16, 17, 20, 23, 31, 33, 35, 38, 39]. Aggarwal et al. [31] reported that the level of injection was at a depth of 3–7 mm, depending upon the size of the lesion. Other terms used for the injection level were “mid-lesion” [12], “body of keloid” [13, 16, 35], “intralesionally” [38], and “into the keloid” [20, 23, 33, 39]. Hietanen et al. [33] specifically mentioned that care was taken not to inject under the keloid mass or too close to the epidermis to avoid local side effects. Khalid et al., Khan et al., and Saha et al. injected the indurated part of a keloid [11, 13, 35]. The angle of injection was described in three studies, being “parallel to the skin” [21], “at 60-degree” [36], and “at 45-degree angle against skin surface” [12]. Nine studies reported administration with multiple injections [11, 13, 17, 18, 23, 31, 33, 35, 38], being separated by 1 cm in six studies [11, 13, 17, 18, 31, 35]. Two studies mentioned the speed of injection, being 0.5–1.0 mL per minute and 0.1 mL per 10–15 s [21, 22]. Blanching was reported in ten (26%) studies as an endpoint indicating adequate TAC distribution [6, 11, 13, 17, 23, 28, 31, 32, 33, 35] (Table 1).

### Study Design

A wide variety of outcome measures was used. The most frequently used outcome measure was keloid size, reported in twelve (34%) studies as either the height, surface area, or volume [19, 20, 23, 27, 29, 30, 31, 32, 35, 38, 40, 41]. Ten (29%) [18, 26, 28, 31, 32, 34, 36, 37, 42, 43] and seven (20%) [6, 7, 12, 25, 33, 44, 45] studies used the Vancouver Scar Scale (VSS) and the Patient and Observer Scar Assessment Scale (POSAS). Remarkably, from 2017 onward, 15 of 22 (68%) studies used either one of these scales. Patient satisfaction was evaluated in eight (23%) studies [17, 19, 22, 27, 28, 30, 41, 42] (Table 2). The treatment results reported by the studies are summarized in online supplementary 3.

The follow-up period in the included studies varied from 1 month [30, 34] to 1 year [29, 35]. Only six (17%) studies had a follow-up of 6 months or longer [7, 19, 23, 29, 35, 41], of which two had a follow-up of 1 year [29] or until recurrence occurred within that year [35]. Eleven (31%) studies did not report the follow-up period. Recurrence was only identified in two studies with a follow-up period of 18 weeks and 1 year [6, 35]. In the latter, the reported recurrence rate was 36% (8 of 22 patients) [35]. In other studies with a minimum follow-up period of 6 months, the recurrence rate was either not specified [7, 19] or zero [23, 29, 41] (Table 2).

Twenty-three (61%) studies reported adverse events. Atrophy (5–75%) and telangiectasia (10–80%) occurred the most and were described in fourteen (37%) studies [13, 17, 18, 25, 28, 30, 31, 33, 37, 40, 41, 42, 44, 45]. The majority (79%) concerned atrophy of the skin. Subcutaneous atrophy was not specified in any study. Four (11%) studies reported that the number of adverse events was zero [14, 19, 22, 27], while in eleven (29%) studies the adverse events were not specified [7, 11, 12, 15, 16, 20, 21, 24, 32, 34, 38] (Table 2).

### Risk-of-Bias Assessment

**Fig. 2:**
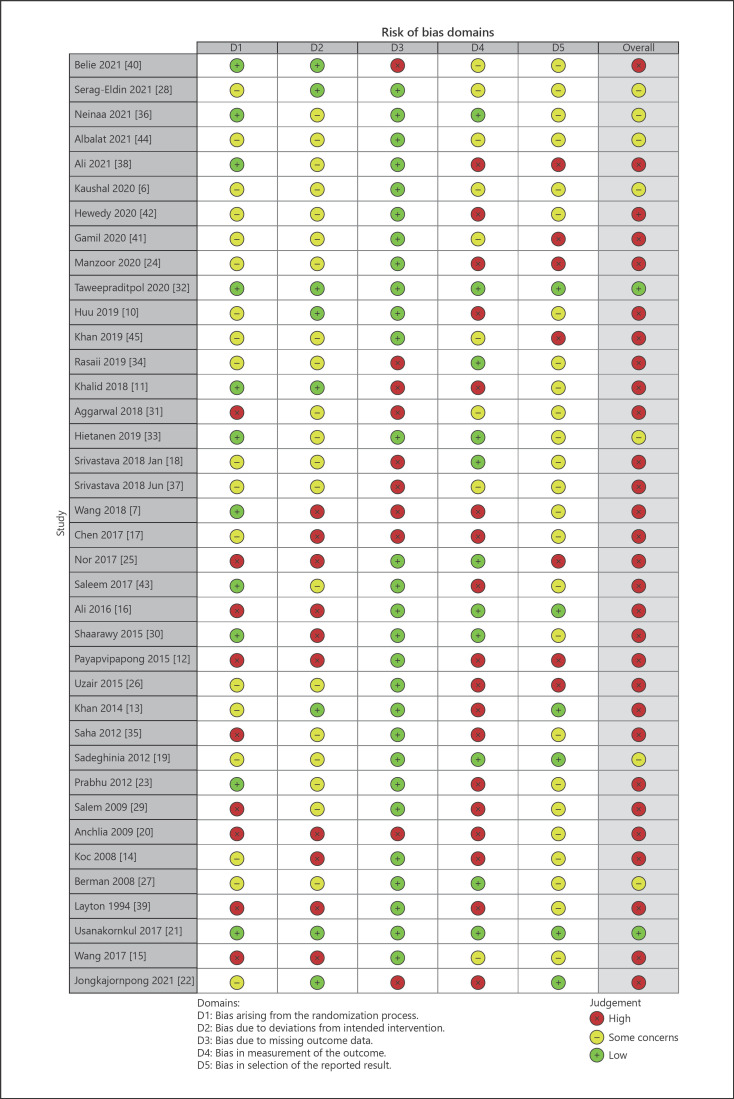
Risk of bias of the included RCTs. The large majority of articles (76%) were judged as having a high risk of bias. Measurement of outcome was most frequently judged as having a high risk of bias (47.4%), followed by deviations from intended intervention (26.3%) and missing outcome data (26.3%), randomization process (23.7%), and selection of reported result (18.4%).

Twenty-nine (76%) articles were judged as having a high risk of bias. The most common reason for high risk of bias was the measurement of outcome (shown in Fig. 2).

## Discussion

This scoping review mapped the relevant details of ICA in keloids described in RCTs. It functions as a baseline measurement of the scientific practice of a first-line treatment for keloids in the best available evidence to date. Incomplete reporting and substantial heterogeneity were identified for different aspects of this pivotal treatment.

### Incomplete Reporting

**Fig. 3:**
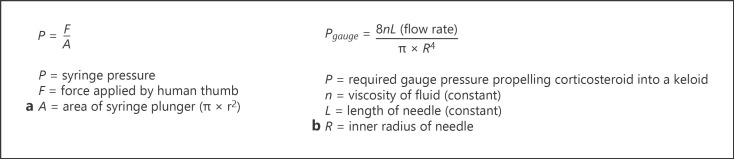
**a** Pascal's law. If the diameter of a syringe plunger doubles or triples, the created pressure using the same force will decrease four and nine times, respectively. **b** Poiseuille's law. The gauge pressure needed to be produced using a 30-gauge needle (inner radius: 0.080 mm) would be approximately six times that of using a 26-gauge needle (inner radius: 0.125 mm) of the same length.

First, the dose of corticosteroid per area of keloid, which is an essential factor that defines treatment effect, could only be extracted from ten (26%) studies. Second, needle (71%) and syringe (89%) sizes were mostly not specified, even though the choice of needle and syringe diameters can influence dose administration. For the syringe, this can be illustrated with Pascal's law (shown in Fig. 3a). Larger syringes generate lower injection pressure that may cause inadequate drug delivery, while small syringes generate high pressure, which increases the risk of drug delivery into the surrounding healthy tissue. Considering needle sizes, Poiseuille's law illustrates that 30-gauge needles are insufficient to deliver adequate medication to firmer keloids (shown in Fig. 3b) [46]. Third, the level of injection is a relevant detail that was reported only by the minority (29%) of studies. Notably, a consensus study of 2019 mentioned that the solid central fibrotic mass of the lesion should not be injected, because the drug will not infiltrate the tissue adequately and the induced rising pressure may cause pain. Instead, it was recommended to penetrate the scar from its border with the normal skin and to inject the deepest part of the scar which is softer than the central core, and/or the periphery of the lesion where the inflammation is particularly pronounced [47]. However, it should be noted that deep injection may increase the risk of subcutaneous atrophy. Considering the endpoint of infiltration, blanching is a way to indicate appropriate drug distribution in the dermis, but was only mentioned in ten (26%) studies.

Apart from the details on ICA, the study design was also incompletely described in most studies. Details on the follow-up period (31%), recurrence rate (66%), and adverse events (29%) were frequently lacking among studies. Only four studies have a follow-up of 6 months or longer, and only two studies have a follow-up 12 months or longer. Long-term powered RCTs are necessary to define treatment outcome. Studies on keloid treatments that do not report long-term treatment effects have little clinical value. Finally, quality assessment of studies revealed a considerable risk of bias in reported treatment results.

### Heterogeneity

TAC is the uniformly used corticosteroid in all but one included study. Different types of corticosteroids exist for intralesional administration. Practical reasons such as drug availability and familiarity with the drug may now be the main reasons for the uniformity in the use of TAC. However, its favorable pharmacokinetic properties may be a more important reason of TAC being the preferred intralesional corticosteroid for keloid treatment originally. Studies have reported that TAC has a substantially longer duration of action in the tissue than betamethasone or prednisolone [48, 49, 50, 51].

Apart from the type of corticosteroid, substantial heterogeneity exists in most other aspects of ICA among included studies. First, TAC doses varied from 1 mg per cm^2^ of keloid [12] to 20 mg per cm^2^ of keloid, a dose difference of 20 times [6, 18, 19]. Second, reported maximum doses per session varied from 20 mg to 80 mg. Systemic adverse events may occur at higher doses of TAC and include Cushing's syndrome. Based on a systematic review of the literature from 1950 to 2012 of reported cases of Cushing's syndrome following intralesional TAC used for the treatment of scars, Fredman et al. [52] recommended that intralesional dosage of TAC should generally not exceed 40 mg per month in adults. Third, treatment sessions varied from one to eight, and treatment intervals varied from weekly to monthly. Ud-Din et al. [53] demonstrated that treatment response is associated with the number of treatment sessions, with an odds ratio of 3.9 (95% CI: 1.06–14.7, *p* = 0.041) for patients receiving more than one session. Treatment interval was studied among sixteen keloids by weekly volume measurements after single TAC injection. Volume reduction was found to be most profound within the first 2 weeks post-injection. This was not significant anymore between 3 and 4 weeks, and a reversal trend was identified after 4 weeks (*p* ≤ 0.05) [54]. In line with this study, the mostly (79%) used treatment interval among RCTs in our study is 3–4 weeks. Furthermore, heterogeneity was identified in needle sizes which varied from 26-gauge (0.45 mm) to 30-gauge (0.30 mm) and the level and angle of injection for which different descriptions were used. Considering local anesthesia, treatment regimens varied from no anesthetics to oral analgesics. Reducing injection pain should be aimed, as pain may affect therapy compliance [55]. Based on the studies that focused on pain relief in ICA, infiltration of the corticosteroid should take place gently, as the speed of drug administration affects pain [21, 22]. Furthermore, local anesthesia using EMLA cream and skin cooling seemed to be effective in attenuating pain [15, 21, 22], while the mixture of an anesthetic with corticosteroid was not [21].

Apart from treatment details, study designs were also very heterogeneous. Outcome measures varied from keloid sizes reported as height, surface area, or volume, to VSS, POSAS, pain and itch scores, patients' satisfaction, and self-defined efficacy rates. The substantial heterogeneity in used outcome measures makes studies incomparable.

### Strengths and Limitations

The value of this scoping review encompasses mapping a first-line treatment for keloids and investigating current research conduct [9]. Even though our scoping review included only RCTs, which are studies that provide the best evidence, overall incomplete reporting and high heterogeneity were identified. The current body of literature is therefore not amenable to data synthesis, and evidence-based treatment recommendation cannot be generated. A limitation of our scoping review is that it included keloids of all sizes, locations, duration, and etiology, even though differences may exist within the same entity [56]. However, differentiation in keloid characteristics could not be made in our study, which is inherent to pooling of data in the best available evidence. In future studies, differentiation between different types of keloids should be considered. Also inherent to available data from current RCTs, the definition of a keloid was frequently not specified, leaving the possibility that other entities such as hypertrophic scars could be included erroneously. Future studies should use a uniform definition, which yet needs to be delineated.

In conclusion, this scoping review highlights the incomplete reporting and substantial heterogeneity on ICA in keloids among current RCTs. Standardization of treatment protocol and study design is urgently needed in order to make future keloid studies and treatment results of ICA comparable.

## Key Message

Incomplete reporting and substantial heterogeneity exist on the intralesional corticosteroid treatment in keloids among current randomized controlled trials.

## Statement of Ethics

It is not applicable because this study is based exclusively on published literature.

## Conflict of Interest Statement

The authors have no conflicts of interest to declare.

## Funding Sources

The authors did not receive funding.

## Author Contributions

Q Yin: conceptualization − equal, data curation − lead, formal analysis − lead, investigation − lead, methodology − lead, project administration − lead, and writing − original draft − lead. JMI Louter: conceptualization − equal, investigation − equal, methodology − supporting, and writing − review and editing − supporting. FB Niessen and O Lapid: supervision − equal, and writing − review and editing − supporting. S Gibss and PPM van Zuijlen: supervision − supporting, and writing − review and editing − supporting. G Tasdemir-Kilic: data curation − equal and investigation − equal. A Wolkerstorfer: conceptualization − lead, methodology − lead, supervision − lead, and writing − review and editing − lead.

## Supplementary Material

Supplementary data

Supplementary data

Supplementary data

## Figures and Tables

**Table 1:** Drug and dosing, equipment, and manual injection technique

Author, year	Country	Patients and keloids that received TAC, *n*	Location (keloids, *n*)	Drug, dose per cm/cm^2^/cm^3^	Volume	Concentration (mg/mL)	Maximum dose per session (mg)	Interval, sessions, *n*	Anesthetics	Needle size, syringe size	Level of injection, angle of injection, injections per session, injection speed, endpoint
Belie et al. (2021) [40]	Nigeria	40 patients, exact number of keloids NS	Head and neck, trunk, upper limb, lower limb. Exact numbers per location NS	NS	NS	NS	NS	Every 2 weeks, 6 sessions	NS	NS	NS

Serag-Eldin et al. (2021) [28]	Egypt	10 patients with 10 keloids	Head and neck (1), chest (1), upper limb (6), lower limb (1), back (1)	NS	NS	20	NS	Every 3 weeks, maximum 5 sessions, or until complete flattening	NS	30-gauge, insulin syringe	Endpoint: lesional blanching

Neinaa et al. (2021) [36]	Egypt	20 patients with 20 keloids	Head and neck (4), trunk (10), extremities (6)	NS	Max. 1 mL	20	20	Every 4 weeks, 3 sessions	NS	Insulin syringe 1 mL (100 units)	At the periphery of keloid Angle of injection: 60°

Albalat et al. (2021) [44]	Egypt	40 patients with >40 keloids, exact number of keloids NS	Below the region of the neck and head. Exact numbers per location NS	NS	NS	40	NS	Every 3 weeks, 6 sessions	NS	NS	NS

Ali et al. (2021) [38]	Pakistan	75 patients with 75 keloid;	NS	NS	0.25 mL	10	NS	Every 3 weeks, 3 sessions	Xylocaine	NS	Intralesionally 2 mm apart

Kaushal et al. (2020) [6]	India	30 patients with 30 keloids	Chest and shoulder. Exact numbers per location for TAC NS	TAC 20 mg per cm^2^	0.5 mL per cm^2^, total 2 mL	40	NS	Every 3 weeks, 6 sessions	NS	Insulin syringe	Endpoint: blanching

Hewedy et al. (2020) [42]	Egypt	20 patients with 20 keloids	Head and neck (2), chest (6), upper limb (9), lower limb (1), back (2)	NS	NS	20	NS	Every 3 weeks, 4 sessions	NS	NS	NS

Gamil et al. (2020) [41]	Egypt	26 patients with >26 keloids, exact number of keloids NS	Ear (10), chest (5), back (3), upper limb (6), lower limb (2)	NS	NS	NS	NS	Every 4 weeks, 3 sessions	NS	NS	NS

Manzoor et al. (2020) [24]	Pakistan	30 patients with 30 keloids	Earlobes, back, shoulders, front of chest. Exact numbers for TAC NS	NS	NS	40	NS	Monthly, 6 sessions	NS	NS	NS

Taweepraditpol (2020) [32]	Thailand	15 patients with 15 keloids	Face (1), ear (1), chest (3), shoulder (4), knee (5), leg (1)	NS	NS	20	NS	Every 4 weeks, 4 sessions	Lidocaine 2% mixed 1:1 with TAC (40 mg/mL) Lidocaine 2% with adrenaline (1:100,000) mixed 1:1 with TAC (40 mg/mL) Local anesthetic cream (lidocaine + prilocaine) 60 min before injection	INS	Endpoint: total pallor of keloid

Huu et al. (2019) [10]	Vietnam	65 patients, exact number of keloids NS	NS	TAC 7.5 or 15 mg per cm^2^	NS	NS	60	Every 4 weeks, on average 4-6 sessions, or until complete flattening	NS	NS	NS

Khan (2019) [45]	Pakistan	82 patients, exact number of keloids NS	Below head and neck region. Exact numbers per location NS	NS	NS	40	NS	Every 4 weeks, 6 sessions	NS	NS	NS

Rasaii (2019) [34]	Iran	23 patients with 23 keloids	NS	NS	Max. 2 mL	20	40	Every 4 weeks, 3 sessions	Local anesthesia	27-gauge	NS

Khalid (2018) [11]	Pakistan	34 patients, exact number of keloids NS	Mostly pre-sternal, head and neck with especially ears. Exact numbers per location NS	NS	NS	NS	NS	Every week, 8 sessions	Xylocaine 1%	27-gauge, syringe 1 mL	On indurated area Multiple pricks separated by 1 cm, for some lesions Endpoint: slight blanching

Aggarwal (2018) [31]	India	16 patients, exact number of keloids NS	Facial keloids excluded. Exact numbers per location for TAC NS	NS	Max. 2 mL	40	80	Every 3 weeks, maximum of 8 sessions, or until complete flattening	NS	30-gauge, insulin syringe	Depth of 3-7 mm At several points, 1 cm apart Endpoint: evenly distributed blanching

Hietanen (2019) [33]	Finland	25 patients with 25 keloids	Chest (6), shoulder (6), upper back (10), abdomen (3)	NS	NS	10	NS	Every 3-4 weeks, 3 sessions	Local anesthesia with lidocaine before lidocaine 1% mixed 1:1 with TAC (20 mg/mL)	NS	Directly into the keloid, not under the keloid mass or too close to epidermis From several directions Endpoint: blanching

Srivastava et al. (2018 Jan) [18]	India	20 patients with 20 keloids	Pre-sternal (11), trunk (5), extremities (3), face (1)	TAC 20 mg per cm^2^	Max. 0.5 mL per cm^2^ Max. 2 mL	40	80	Every 3 weeks, 8 sessions, or until keloid resolved	Oral analgesics	27-gauge, insulin syringe	Multiple pricks, 1 cm apart

Srivastava (2018 Jun) [37]	India	20 patients with 20 keloids	Pre-sternal (8), trunk (6), extremities (4), face (2)	NS	Max. 2 mL	40	80	Every 3 weeks, 8 sessions, or until scar flattening	NS r	Insulin syringe	NS

Wang et al. (2018) [7]	Taiwan	17 patients with 17 keloids	Extremity (17)	TAC 10 mg per cm	1 mL per cm	10	NS	Every 2 weeks, 3 sessions	NS	NS	NS

Chen (2017) [17]	China	Exact number of patients and keloids NS (±23)	Exact numbers for TAC NS	Betamethasone disodium phosphate 1 mg and betamethasone dipropionate 2.5 mg per cm^2^ at maximum	Modified to degree of lesions Max. 0.5 mL per cm^2^ Max. final dosage: 2 mL	2 5	4 10	Monthly, 3 sessions	NS	Syringe needle with diameter 0.45 mm	Tough portion of keloid Several injections, separated by approx. 1 cm Endpoint: slight blanching

Nor et al. (2017) [25]	Malaysia	21 patients with 21 keloids	Shoulders (10), chest (7), forearm (2), stomach (2)	NS	NS	40	NS	Monthly	NS	NS	NS

Saleem (2017) [43]	Pakistan	50 patients, number of keloids NS	NS	NS	0.1	40	NS	Every 4 weeks, 3 sessions, or until flattening	NS	NS	NS

Ali (2016) [16]	Pakistan	19 keloids, number of patients NS	NS	NS	NS	10	NS	Weekly, 8 sessions	NS	27-gauge, syringe 1 mL	Body of scar

Shaarawy et al. (2015) [30]	Egypt	12 patients, exact number of keloids NS	NS	NS	NS	10	NS	Every 4 weeks, 6 sessions, or until complete improvement	NS	NS	NS

Payapvipapong (2015) [12]	Thailand	3 patients with 3 keloids	Abdomen, back, chest, extremities. Exact numbers per location for keloids NS	TAC 1 mg per cm^2^	0.1 mL per cm^2^ Max. 6 mL	10	60	Every 4 weeks, 3 sessions	NS	27-gauge	Mid-lesion Angle of injection: 45°, bevel up

Uzair et al. (2015) [26]	Pakistan	40 patients with 40 keloids	NS	NS	1 mL	40	NS	Monthly, maximum of 3 sessions, or until keloid flattened	NS	NS	NS

Khan et al. (2014) [13]	Pakistan	33 patients with 33 keloids	NS	NS	Max. 2 mL	10	20	Every week, 8 sessions	Xylocaine 1%	NS	Body of scar, indurated part Multiple injections separated by 1 cm, if needed Endpoint: slight blanching

Saha (2012) [35]	India	24 patients with >24 keloids, exact number of keloids NS	Upper aspect of the back, chest, and arms Exact numbers per location NS.	NS	Max. 2 mL 0.2-0.4 mL/cm^2^	40	80	Every week, 6 sessions, or until satisfactory effect (average: 4)	NS	30-gauge, insulin syringe 1 mL	Body of keloid Indurated, firm portion Multiple injections separated by approx. 1 cm Endpoint: slight blanching

Sadeghinia (2012) [19]	Iran	Exact number of patients and keloids NS (±20)	Face and neck, trunk, upper and lower limbs. Exact numbers per location for TAC NS	TAC 20 mg per cm^2^	0.5 mL per cm^2^	40	NS	Every 4 weeks, 3 sessions	Lidocaine 2%, before TAC	27-gauge	NS

Prabhu et al. (2012) [23]	India	15 patients with 15 keloids	Shoulder (7), upper limb (1), chest (6), back (1)	NS	Max. 2 mL	40	80	Weekly, 4 sessions	NS	Insulin syringe	Into the substance of keloid At multiple sites on keloid, solution pushed with adequate pressure Endpoint: minimal blanching

Salem et al. (2009) [29]	Egypt	10 patients with 10 keloids	Neck, face, breast, forearm, earlobe, mastoid region, deltoid region. Exact numbers per location for TAC NS	NS	NS	20	NS	Every 3 weeks, maximum 6 sessions, or until clearance	NS	NS	NS

Anchlia et al. (2009) [20]	India	11 patients, exact number of keloids NS	NS	TAC 4 mg per cm^2^	0.1 mL per cm^2^ Max. 10 cm^2^	40	40	1 session	NS	Insulin syringe	Directly into the keloid

Koc et al. (2008) [14]	Turkey	9 patients with 9 keloids	Head or neck (2), upper extremity (4), thorax (7)	NS	Different because of varying sizes	40	NS	Every 4 weeks, 4 sessions	NS	27-gauge	NS

Berman et al. (2008) [27]	USA	9 patients, exact number of keloids NS	NS	NS	NS	20	NS	Monthly, 2 sessions	NS	NS	NS

Layton et al. (1994) [39]	UK	11 patients with multiple acne keloids, exact number of keloids NS	Face, back, chest. Exact numbers per location NS	NS	1 mL	5	NS	NS	NS	NS	Into the keloid

Usanakornkul (2017) [21]	Thailand	40 patients with 40 keloids	Ear lobule (10), shoulders (18), and sternum (18)	TAC 10 mg per cm^3^	1 mL per cm^3^	10	NS	Every 3 weeks, patients received all 4 methods	2-mm-thick EMLA 1 h before treatment with lidocaine 1% mixed 1:1 with TAC	NS	1 injection during 1 visit Direction of the needle: parallel to skin Speed of injection: 0.5-1.0 mL/min

Wang et al. (2017) [15]	China	17 patients with 21 keloids	Chest (9), shoulder (3), back (1), arm (3), mandible (1)	NS	NS	NS	NS	Monthly	CryoTip device (−10 °C) for 15 s before TAC	NS	NS

Jong kajorn pong (2021) [22]	Thailand	17 patients with 21 keloids	Ear (10), neck (1), back (2), chest (10), abdomen (5), upper extremities (11), lower extremities (4)	TAC 10 mg per cm^(2)^	1 mL per cm^(2)^	10	NS	Every 3-5 weeks	Skin cooling with ice pack for 3 min EMLA cream, covered with film, 60 min before injection	NS	Speed of injection: 0.1 mL per 10-15 s

Table 1 presents (1) drug- and dosing-related data including type, volume, and concentration of the used corticosteroid, the calculated dose per cm, cm^2^, or cm^3^, the maximum dose per session (mg) as calculated by the used maximum volume and concentration of corticosteroid, and the use of local anesthetics, (2) equipment-related data including the needle and syringe size, and (3) manual injection technique-related data including the level of injection, angle of injection, speed of injection, number of injections per session, and endpoint of infiltration. NS, not specified.

**Table 2:** Study design

Author, year, country	Patients and keloids that received TAC, *n*	Location (keloids, *n*)	Outcome measure(s)	Follow-up (F), recurrence (R)	Adverse events
Belie et al. (2021) [40], Nigeria	40 patients, number of keloids NS	Head and neck, trunk, upper limb, lower limb	Length, width, height Pain score Pruritus score	F: 3 months R: NS	Skin atrophy and hypopigmentation: 75% Ulceration: 5% Pain following drug injection: number NS

Serag-Eldin et al. (2021) [28], Egypt	10 patients with 10 keloids	Head and neck (1), chest (1), upper limb (6), lower limb (1), back (1)	VSS VRS pain and itch Patient satisfaction	F: 3 months R: none	Atrophy: 60% Hypopigmentation: 60% Telangiectasia: 80% TAC precipitations: 70% Striae: 10%

Neinaa et al. (2021) [36], Egypt	20 patients with 20 keloids	Head and neck (4), trunk (10), extremities (6)	VSS Clinical efficacy, defined by degree of VSS improvement Dermoscopic examination Histopathological and immunohistochemical assessments	F: NS R: none	Hypopigmentation: 20%, after the third treatment session

Albalat et al. (2021) [44], Egypt	40 patients with >40 keloids	Below the region of the neck and head	POSAS Efficacy, defined >50% decrease of POSAS	F: 6 weeks R: none	Hypopigmentation: 70% Telangiectasia: 20% Pain: 100%

Ali et al. (2021) [38], Pakistan	75 patients with 75 keloids	NS	Mean size (mm) Efficacy, defined >75% reduction in keloid size	F:±2 weeks R: NS	NS

Kaushal et al. (2020) [6], India	30 patients with 30 keloids	Chest and shoulder	POSAS Grade of improvement defined by reduction in POSAS score	F: 18 weeks R: 6 of 30 patients, from 27 weeks onward	Hypopigmentation: 16.6% Pain: 13.3%

Hewedy et al. (2020) [42], Egypt	20 patients with 20 keloids	Head and neck (2), chest (6), upper limb (9), lower limb (1), back (2)	VSS VRS pain VRS itch Patient satisfaction	F: 3 months R: NS	Atrophy: 35% Hypopigmentation: 50% Telangiectasia: 50% TAC precipitations: 80%

Gamil et al. (2020) [41], Egypt	26 patients with >26 keloids	Ear (10), chest (5), back (3), upper limb (6), lower limb (2)	Stony Brook Scar Evaluation Scale, i.e., improvement of width, height, color, suture marks, overall appearance Color Doppler ultrasound Patient satisfaction	F: 6 months R: none	Skin atrophy: 7.7% Pain du ring injection: 11.5%

Manzoor et al. (2020) [24], Pakistan	30 patients with 30 keloids	Earlobes, back, shoulders, front of chest	Efficacy, defined 51-100% improvement of flattening and decrease in size of lesion	F: none R: NS	NS

Taweepraditpol (2020) [32], Thailand	15 patients with 15 keloids	Face (1), ear (1), chest (3), shoulder (4), knee (5), leg (1)	Volume reduction VSS VAS pain	F: 4 weeks R: NS	NS

Huu et al. (2019) [10], Vietnam	65 patients, number of keloids NS	NS	Clinical evaluation criteria of Henderson (1998) and El-Tonsy (1996), based on height, stiffness, color, symptoms, recurrence, and side effects	F: NS R: NS	7.5 mg/cm^2^: ulcers - 3.0%. Menstrual disorders: 5.6%. Hypertension: 3.0% 15 mg/cm^2^: ulcers - 18.6%. Acnes: 6.4%. Menstrual disorders: 25%. Hypertension: 3.1%

Khan et al. (2019) [45], Pakistan	82 patients, number of keloids NS	Below head and neck region	POSAS Efficacy, defined >50% reduction POSAS compared to baseline	F: NS R: NS	Skin atrophy: 70% Hypopigmentation: 29% Telangiectasias: 21%

Rasaii et al. (2019) [34], Iran	23 patients, with 23 keloids	NS	VSS VAS pain. VAS pruritus	F: 1 month R: NS	NS

Khalid et al. (2018) [11], Pakistan	34 patients, number of keloids NS	Mostly pre-sternal, head and neck with especially ears	Efficacy defined as >50% reduction in height Height, patient and observer assessment scale for height reduction	F: NS for keloids R: NS for keloids	NS for keloids

Aggarwal et al. (2018) [31], India	16 patients, number of keloids NS	Facial keloids excluded	Clearance defined as reduction in height of keloid to <1 mm Height, VSS VAS patient, VAS doctor	F: none R: NS	Atrophy depigmentation: 31.25% Telangiectasia: 31.25% Overall side effects: 50%

Hietanen et al. (2019) [33], Finland	25 patients with 25 keloids	Chest (6), shoulder (6), upper back (10), abdomen (3)	Remission defined as keloid flattening where no injections were feasible or needed POSAS Hemoglobin concentration, blood vessel density, fibroblast proliferation	F: none R: NS	Skin atrophy: 44% Telangiectasia: 50%

Srivastava et al. (2018 Jan) [18], India	20 patients with 20 keloids	Pre-sternal (11), trunk (5), extremities (3), face (1)	VSS VRS pain and pruritus	F: final evaluation 30 weeks after first dose R: none	Skin atrophy: 20% Telangiectasia: 15% Pain at injection site: 24%

Srivastava et al. (2018 Jun) [37], India	20 patients with 20 keloids	Pre-sternal (8), trunk (6), extremities (4), face (2)	VSS	F: NS R: NS	Skin atrophy: 5% Telangiectasia: 10% Pain at injection site: 40%

Wang et al. (2018) [7], Taiwan	17 patients with 17 keloids	Extremity (17)	POSAS, VRS pain, itching Blood perfusion scan, Masson trichrome stain, collagen I, collagen II, collagen III, collagen X Angiogenesis: VEGF, CD31. Inflammatory cytokines: TGF-ß1, IL-6 Apoptosis: MMP-13, TUNEL. Proliferation: PCNA, fibronectin, MMP-13	F:48 weeks R: NS	NS

Chen et al. (2017) [17], China	Number of patients and keloids NS (±23)	NS	Patient self-assessment, improvement ≥50% Observer assessment, improvement >50% Reduction in erythema, toughness, and pruritus	F: none R: NS	Skin atrophy and telangiectasia: 36% Almost all injections were painful

Nor et al. (2017) [25], Malaysia	21 patients with 21 keloids	Shoulders (10), chest (7), forearm (2), stomach (2)	POSAS VAS pain Patient preferring	F: NS R: NS	Skin atrophy: 23.5% Hypopigmentation: 35.3% Telangiectasia: 41.2% Pain: 100%. Erythema: 41.2%. Bleeding: 17.6%. Cutaneous necrosis: 70.6%

Saleem et al. (2017) [43], Pakistan	50 patients, number of keloids NS	NS	VSS Efficacy	F: 12 weeks R: none	Pain at time of injection, number NS

Ali et al. (2016) [16], Pakistan	19 keloids, number of patients NS	NS	Efficacy defined >50% reduction in initial keloid in terms of observer scar, absence of all complications (i.e., skin atrophy, hypopigmentation, telangiectasias, and skin ulcerations)	F:4 weeks R: NS	NS

Shaarawy et al. (2015) [30], Egypt	12 patients, number of keloids NS	NS	Volume (cm^3^). Height (scale: 0-3) Hardness (scale: 0-3). Redness (scale: 0-3) Itching (scale: 0-3). Pain (scale: 0-3). Tenderness (scale: 0-3). Patient satisfaction	F: 1 month R: NS	Skin atrophy and telangiectasia: 25% Mild pain or discomfort during and few hours after the procedure, number NS

Payapvipapong et al. (2015) [12], Thailand	3 patients with 3 keloids	Abdomen, back, chest, extremities	POSAS Patient satisfaction Scar thickness	F: NS for keloids R: NS	NS for keloids

Uzair et al. (2015) [26], Pakistan	40 patients with 40 keloids	NS	VSS	F: 3 months R: none	Hypopigmentation: 12.5% Pain: almost all patients, number NS Irregular menstrual cycles: 5%

Khan et al. (2014) [13], Pakistan	33 patients with 33 keloids	NS	Effectivity defined >50% reduction in initial scar height	F: 4 weeks to a maximum of 6 month; R: none	Skin atrophy and telangiectasia: 30.30%

Saha (2012) [35], India	24 patients with >24 keloids	Upper aspect of the back, chest, arms	Reduction of volume Reduction in pain Reduction in itching	F: 1 year or until recurrence R: 8 of 22 (36.36%) <6 months of last treatment	Hyperpigmentation: 12.5% Pain at injection site: 4.17%

Sadeghinia (2012) [19], Iran	Number of patients and keloids NS (±20)	Face and neck, trunk, upper and lower limbs	Height (mm). Surface (mm^2^) Erythema (5-point scale). Induration (5-point scale) Pruritus (5-point scale). Patient self-assessment. Observer assessment	F: 32 weeks R: NS	None

Prabhu et al. (2012) [23], India	15 patients with 15 keloids	Shoulder (7), upper limb (1), chest (6), back (1)	Volume VAS pain Consistency Clinical appearance (atrophic, hypertrophic, nodular)	F: 6 months R: none	Increased pruritus: 6.7%

Salem et al. (2009) [29], Egypt	10 patients with 10 keloids	Neck, face, breast, forearm, earlobe, mastoid region, deltoid region	Flattening and size reduction VEGF expression	F: 1 year R: none	Pain: 40% Hypertrichosis: 20%

Anchlia et al. (2009) [20], India	11 patients, number of keloids NS	NS	Volume Overall response to therapy (%)	F: 3 months R: NS	NS

Koc et al. (2008) [14], Turkey	9 patients with 9 keloids	Head or neck (2), upper extremity (4), thorax (7)	NS for keloids	F: 2 months R: NS	None

Berman et al. (2008) [27], USA	9 patients, number of keloids NS	NS	Lesion size Lesion induration, erythema, pigmentary alteration, pain, pruritus Cosmetic assessment: investigator, patient Patient satisfaction scale	F: 4 weeks R: NS	None

Layton et al. (1994) [39], UK	11 patients, number of keloids NS	Face, back, chest	Degree of response (%)	F: 8 weeks R: NS	Discomfort at the time of administration

Usanakornkul (2017) [21], Thailand	40 patients with 40 keloids	Ear lobule (10), shoulders (18), sternum (18)	VAS pain Pain relief duration	NA	NS

Wang et al. (2017) [15], China	17 patients with 21 keloids	Chest (9), shoulder (3), back (1), arm (3), mandible (1)	VAS pain Verbal descriptor scale	NA	NS

Jong kajorn pong (2021) [22], Thailand	17 patients with 21 keloids	Ear (10), neck (1), back (2), chest (10), abdomen (5), upper extremities (11), lower extremities (4)	VAS pain	NA	None for ice packing

Table 2 presents study design-related data including outcome measures, follow-up period, recurrence, and adverse events. NS, not specified; NA, not applicable; VSS, Vancouver Scar Scale, POSAS, Patient and Observer Scar Assessment Scale; VRS, verbal rating scale; IQR, interquartile range; SEM, standard error of the mean. *Data difficult to acquire from graph or diagram.
