# Supplementary material for: Intralesional Corticosteroid Administration in the Treatment of Keloids: A Scoping Review on Injection Methods
Source: Dermatology. 2023 Jan 19;239(3):462–77. doi: 10.1159/000529220 (PMC10906477; doi:10.1159/000529220)
Supplement: Supplementary file 2 — Supplementary data [file drm-0239-0462-s02.docx]

**Supplement 1. Detailed search terms**

The exact search term combinations used in PubMed, Ovid MEDLINE, Ovid EMBASE and CENTRAL.

PubMed:

((Keloid[MeSH Terms]) OR (Keloid*[Title/Abstract])) AND (inject*[Title/Abstract] OR intralesion*[Title/Abstract] OR injection, intralesional[MeSH Terms] OR injections, intralesional[MeSH Terms])

Ovid MEDLINE:

(exp Keloid/ or keloid*.ti,ab,kw.) and (exp Injections/ or exp Injections, Intralesional/ or inject*.ti,ab,kw. or intralesion*.ti,ab,kw. or infiltrat*.ti,ab,kw.)

Ovid EMBASE:

(exp keloid/ or keloid*.ti,ab,kw.) and (exp injection/ or exp intralesional drug administration/ or inject*.ti,ab,kw. or intralesion*.ti,ab,kw. or infiltrat*.ti,ab,kw.)

CENTRAL:

#1: MeSH descriptor: [Keloid] explode all trees

#2: MeSH descriptor: [Injections] explode all trees
#3: (inject* or intrales* or infiltrate*):ti,ab,kw

#4: (keloid*):ti,ab,kw

#5: (#1 or #4) and (#2 or #3)
