# Supplementary material for: Intralesional Corticosteroid Administration in the Treatment of Keloids: A Scoping Review on Injection Methods
Source: Dermatology. 2023 Jan 19;239(3):462–77. doi: 10.1159/000529220 (PMC10906477; doi:10.1159/000529220)
Supplement: Supplementary file 3 — Supplementary data [file drm-0239-0462-s03.docx]

Supplement 3. Study design and treatment results

| **Author, year, country** | **Number of patients and keloids that received TAC** | **Location (number of keloids)** | **Outcome measure(s) and treatment result(s)** | **Follow-up (F), recurrence (R)** | **Adverse events** |
| --- | --- | --- | --- | --- | --- |
| Belie 2021 [40], Nigeria | 40 patients, number of keloids NS | Head and neck, trunk, upper limb, lower limb | Length, width, height: decrease in mean width and mean height by the third clinic visit*  P values, mean pain score at 3-month follow-up vs. presentation:  head/neck: 0.017, trunk: 0.027, upper limb: 0.109  P values, mean pruritus score at 3-month follow-up vs. presentation: head/neck: 0.002, trunk: 0.014, upper limb: 0.109, lower limb: 0.083 | F: 3 months  R: NS | Skin atrophy and hypopigmentation: 75%  Ulceration: 5%  Pain following drug injection: number NS |
| Serag-Eldin 2021 [28], Egypt | 10 patients with 10 keloids | Head and neck (1), chest (1), upper limb (6), lower limb (1), back (1) | VSS: improvement in 65.1% ± 14.8  VRS pain and itch: improvement*  Patient satisfaction: 70% satisfied | F: 3 months  R: None | Atrophy: 60%  Hypopigmentation: 60%  Telangiectasia: 80%  TAC precipitations: 70%  Striae: 10% |
| Neinaa 2021 [36], Egypt | 20 patients with 20 keloids | Head and neck (4), trunk (10), extremities (6) | Mean VSS after treatment: 5.0 ± 2.4 (baseline: 8.8 ± 2.7), p < 0.001  Clinical efficacy, defined by degree of VSS improvement: none (0%), 1-25% (65%), 50-75% (25%), >75% (10%)  VRS pain and itch after treatment: p = 0.01, p = 0.001  Dermoscopic examination: moderate reduction in vascular pattern  Histopathological and immunohistochemical assessments: reduction of collagen deposition, reduction of CTGF immune expression | F: NS  R: None | Hypopigmentation: 20%, after the third treatment session |
| Albalat 2021 [44], Egypt | 40 patients with >40 keloids | Below the region of the neck and head | POSAS after 24 weeks: 36 ± 12.74 (baseline: 91 ± 10.98)  Efficacy, defined >50% decrease of POSAS: 75% | F: 6 weeks  R: None | Hypopigmentation: 70%  Telangiectasia: 20%  Pain: 100% |
| Ali 2021 [38], Pakistan | 75 patients with 75 keloids | NS | Mean size (mm) at week 14: 4.36 ± 2.81 (baseline: 8.48 ± 3.08) Efficacy, defined >75% reduction in keloid size: 36% | F: ± 2 weeks  R: NS | NS |
| Kaushal 2020 [6], India | 30 patients with 30 keloids | Chest and shoulder | Mean POSAS after 33 weeks: 15.43 (baseline: 63.20 ± 23.134)  Grade of improvement defined by reduction in POSAS score: none (0%), <25% (0%), 25-50% (13.3%), 51-75% (13.3%), >75% (73.4%) | F: 18 weeks  R: 6 of 30 patients, from 27 weeks onwards | Hypopigmentation: 16.6%  Pain: 13.3% |
| Hewedy 2020 [42], Egypt | 20 patients with 20 keloids | Head and neck (2), chest (6), upper limb (9), lower limb (1), back (2) | VSS after 3 months of follow-up: 1.95 ± 1.84 (before treatment: 6.95 ± 2.4)  VRS pain, improvement: 88.32 ± 22.15  VRS itch, improvement: 82.54 ± 20.75  Patient satisfaction: 55% satisfied | F: 3 months  R: NS | Atrophy: 35%  Hypopigmentation: 50%  Telangiectasia: 50%  TAC precipitations: 80% |
| Gamil 2020 [41], Egypt | 26 patients with >26 keloids | Ear (10), chest (5), back (3), upper limb (6), lower limb (2) | Subjective assessment using Stony Brook Scar Evaluation Scale, i.e. improvement of width, height, color, suture marks, overall appearance (p = 0.0001)  Objective assessment using Color Doppler Ultrasound, improvement after treatment: thickness (42.6%), transverse axis (63.2%), longitudinal axis (16%), surface area (68%), vascularity, calcifications, invasion of muscle wall, invasion of hypodermis  Patient satisfaction: 85% (highly) satisfied | F: 6 months  R: None | Skin atrophy: 7.7%  Pain during injection: 11.5% |
| Manzoor 2020 [24], Pakistan | 30 patients with 30 keloids | Earlobes, back, shoulders, front of chest | Efficacy, defined 51-100% improvement of flattening and decrease in size of lesion: 70% | F: none  R: NS | NS |
| Taweepraditpol 2020 [32], Thailand | 15 patients with 15 keloids | Face (1), ear (1), chest (3), shoulder (4), knee (5), leg (1) | Volume reduction (mL) measured with molding and water replacement method: TAC: 0.34 ± 0.52 (47.95%), TAC with lidocaine: 0.41 ± 0.43 (62.1%), TAC with lidocaine with adrenaline: 0.53 ± 0.93 (42.07%) (p = 0.650)  VSS, better vs. same or worse after treatment (number of patients):  Vascularity: TAC: 2/3, TAC with lidocaine: 2/3, TAC with lidocaine with adrenaline: 4/1 (p = 0.53)  Pigmentation: TAC: 1/4, TAC with lidocaine: 0/5, TAC with lidocaine with adrenaline: 1/1 (p = 0.1)  Pliability: TAC: 2/3, TAC with lidocaine: 5/0, TAC with lidocaine with adrenaline: 2/1 (p = 0.25)  Height: TAC: 2/3, TAC with lidocaine: 2/3, TAC with lidocaine with adrenaline: 4/1 (p = 1.0)  VAS pain: TAC: 4/1, TAC with lidocaine: 5/0, TAC with lidocaine with adrenaline: 2/1 (p = 1.0) | F: 4 weeks  R: NS | NS |
| Huu 2019 [10], Vietnam | 65 patients, number of keloids NS | NS | Clinical evaluation criteria of Henderson (1998) and El-Tonsy (1996), based on height, stiffness, color, symptoms, recurrence and side effects:  7.5 mg/cm^2^: good: 24.2%, quite good: 66.7%, poor: 9.1%. Flattened scars: 78.8%  15 mg/cm^2^: good: 15.6%, quite good: 53.1%, poor: 31.3%. Flattened scars: 65.6% | F: NS  R: NS | 7.5 mg/cm^2^:  Ulcers: 3.0%  Menstrual disorders: 5.6%  Hypertension: 3.0%  15 mg/cm^2^:  Ulcers: 18.6%  Acnes: 6.4%  Menstrual disorders: 25%  Hypertension: 3.1% |
| Khan 2019 [45], Pakistan | 82 patients, number of keloids NS | Below head and neck region | POSAS after 24 weeks: 34 ± 12.28 (baseline: 90 ± 10.85)  Efficacy, defined >50% reduction POSAS compared to baseline: 70% | F: NS  R: NS | Skin atrophy: 70%  Hypopigmentation: 29%  Telangiectasias: 21% |
| Rasaii 2019 [34], Iran | 23 patients, with 23 keloids | NS | After 1 month of follow-up:  Mean VSS: 4.04 SEM 0.31 (baseline: 6.43 SEM 0.37), p < 0.001  Mean VAS pain: 2.61 SEM 0.21 (baseline: 3.91 SEM 0.33), p = 0.001  Mean VAS pruritus: 1.30 SEM 0.17 (baseline 2.00 SEM 0.17), p = 0.02 | F: 1 month  R: NS | NS |
| Khalid 2018 [11], Pakistan | 34 patients, number of keloids NS | Mostly pre-sternal, head and neck with especially ears | Efficacy defined as >50% reduction in height, 4 weeks after end of treatment: 44.1%  Mean reduction in height, patient and observer assessment scale for height reduction: NS for keloids | F: NS for keloids  R: NS for keloids | NS for keloids |
| Aggarwal 2018 [31], India | 16 patients, number of keloids NS | Facial keloids excluded | Clearance defined as reduction in height of keloid to ≤1 mm: 75%  Change in mean from baseline: height: 3.18, VSS: 4.12, VAS patient: 7.82, VAS doctor: 8.18 | F: none  R: NS | Atrophy-depigmentation: 31.25%  Telangiectasia: 31.25%  Overall side effects: 50% |
| Hietanen 2019 [33], Finland | 25 patients with 25 keloids | Chest (6), shoulder (6), upper back (10), abdomen (3) | At 6 months after treatment:  Remission defined as flattening of the keloid to the degree where no further injections were feasible or needed: 60%  POSAS decreased*, p < 0.05  Estimated hemoglobin concentration: 0.10 ± 0.18 (baseline: 0.17 ± 0.18), p < 0.05  Blood vessel density decreased*, p < 0.05  Fibroblast proliferation: 7.9%, range 1.7-24.7 (baseline: 11.3%, range 1.9-25.3), p < 0.05 | F: none  R: NS | Skin atrophy: 44%  Telangiectasia: 50% |
| Srivastava 2018 Jan [18], India | 20 patients with 20 keloids | Pre-sternal (11), trunk (5), extremities (3), face (1) | VSS scores 30 weeks after first dose:  Mean height: 0.61 ± 0.45 (baseline: 1.7 ± 0.57)  Mean vascularity: 0.15 ± 0.37 (baseline: 1.85 ± 0.37)  Mean pliability: 0.75 ± 0.44 (baseline: 2.8 ± 0.41)  Mean pigmentation: 0.7 ± 0.47 (baseline: 1.85 ± 0.37)  VRS scores 30 weeks after first dose:  Mean pain: 0 ± 0 (baseline: 2.05 ± 0.89)  Mean pruritus: 0.30 ± 0.45 (baseline: 2.75 ± 0.44) | F: final evaluation 30 weeks after first dose  R: None | Skin atrophy: 20%  Telangiectasia: 15%  Pain at injection site: 24% |
| Srivastava 2018 Jun [37], India | 20 patients with 20 keloids | Pre-sternal (8), trunk (6), extremities (4), face (2) | VSS scores at 24 weeks:  Mean height: 0 ± 0 (baseline: 1.75 ± 0.64)  Mean vascularity: 0 (baseline: 1.65 ± 0.49)  Mean pliability: 0 ± 0 (baseline: 1.9 ± 0.64)  Mean pigmentation: 0.8 ± 0.41 (baseline: 1.7 ± 0.47) | F: NS  R: NS | Skin atrophy: 5%  Telangiectasia: 10%  Pain at injection site: 40% |
| Wang 2018 [7], Taiwan | 17 patients with 17 keloids | Extremity (17) | After treatment (before treatment):  POSAS patient: 20.0 ± 5.4 (36.9 ± 6.7), p < 0.01  POSAS observer: 18.0 ± 5.6 (33.2 ± 7.3), p < 0.01  VRS pain score: 0.4 ± 0.9 (1.4 ± 1.5), p = 0.022  Itching: mild: 16 (5), moderate: 1 (8), severe: 0 (4)  Blood perfusion scan: 0.01 ± 0.02 (0.02 ± 0.02), p = 0.095  Masson Trichrome stain: 85.66 (89.5)  Collagen I: 19.31 (22.63), collagen II: 19.31 (22.63), collagen III: 17.04 (21.58) p < 0.05, collagen X: 24.43 (26.72)  Angiogenesis: VEGF 34.4 (31.94), CD31 19.78 (19.54)  Inflammatory cytokines: TGF-β1 29.31 (37.63), IL-6 22.83 (27.44)  Apoptosis: MMP-13 13.61 (11.01) p < 0.05, TUNEL 51.79 (34.24) p < 0.001  Proliferation: PCNA 41.26 (44.99), fibronectin 19.49 (25.49) p < 0.001, MMP-13 13.61 (11.01) p < 0.05 | F: 48 weeks  R: NS | NS |
| Chen 2017 [17], China | Number of patients and keloids NS (±23) | NS | At three months:  Patient self-assessment, improvement ≥50%: 20%  Observer assessment, improvement ≥50%: 12%  Reduction in erythema, toughness and pruritus: * statistically significant (p < 0.05) | F: None  R: NS | Skin atrophy and telangiectasia: 36%  Almost all injections were painful |
| Nor 2017 [25], Malaysia | 21 patients with 21 keloids | Shoulders (10), chest (7), forearm (2), stomach (2) | At 12 weeks:  POSAS observers: 23.50 (baseline 36.00), p = 0.0005  POSAS patients: 16.00 (baseline: 31.00), p = 0.001  Median VAS pain: 5.8, IQR 3 (range 3-10)  Patient survey: 47% prefer intralesional TAC | F: NS  R: NS | Skin atrophy: 23.5%  Hypopigmentation: 35.3%  Telangiectasia: 41.2%  Pain: 100%  Erythema: 41.2%  Bleeding: 17.6%  Cutaneous necrosis: 70.6% |
| Saleem 2017 [43], Pakistan | 50 patients, number of keloids NS | NS | After treatment (before treatment):  VSS: 4.82 ± 1.12 (9.80 ± 1.56), p = 0.001  Efficacy: 62% | F: 12 weeks  R: None | Pain at time of injection, number NS |
| Ali 2016 [16], Pakistan | 19 keloids, number of patients NS | NS | 4 weeks after end of treatment:  Efficacy defined > 50% reduction in initial keloid in terms of observer scar assessment (size measured by scale), absence of all complications (i.e. skin atrophy, hypopigmentation, telangiectasias and skin ulcerations): 21.05% | F: 4 weeks  R: NS | NS |
| Shaarawy 2015 [30], Egypt | 12 patients, number of keloids NS | NS | 7 months after treatment (baseline)  Volume (cm^3^): 0.77 ± 0.749 (4.09 ± 1.830), volume reduction: 82.7%, p < 0.01  Hardness (scale: 0-3): 0.167 ± 0.389 (3 ± 0), p < 0.01  Height (scale: 0-3): 0.83 ± 0.937 (2.92 ± 0.289), p < 0.01  Redness (scale: 0-3): 0.58 ± 0.668 (2.83 ± 0.389), p < 0.01  Itching (scale: 0-3): 0.92 ± 0.668 (2.67 ± 0.492), p < 0.01  Pain (scale: 0-3): 1 ± 0.738 (2.67 ± 0.651), p < 0.01  Tenderness (scale: 0-3): 1.25 ± 0.621 (2.5 ± 0.674), p < 0.01  Patient satisfaction: 50% highly satisfied, 42% satisfied, 8% unsatisfied | F: 1 month  R: NS | Skin atrophy and telangiectasia: 25%  Mild pain or discomfort during and few hours after the procedure, number NS |
| Payapvipapong 2015 [12], Thailand | 3 patients with 3 keloids | Abdomen, back, chest, extremities | POSAS, numbers NS for keloids  Patient satisfaction, numbers NS for keloids  Photographic evaluation, numbers NS for keloids  Scar thickness measured by ultrasonography, numbers NS for keloids | F: NS for keloids  R: NS | NS for keloids |
| Uzair 2015 [26], Pakistan | 40 patients with 40 keloids | NS | Mean decrease VSS after completion of treatment: 4.35 ± 0.700, 58.28% reduction in baseline score | F: 3 months  R: None | Hypopigmentation: 12.5%  Pain: almost all patients, number NS  Irregular menstrual cycles: 5% |
| Khan 2014 [13], Pakistan | 33 patients with 33 keloids | NS | Effectivity defined >50% reduction in initial scar height: 60.60% | F: 4 weeks to a maximum of 6 months  R: None | Skin atrophy and telangiectasia: 30.30% |
| Saha 2012 [35], India | 24 patients with >24 keloids | Upper aspect of the back, chest, arms | Reduction of volume: 76-100%: 12.5%, 51-75%: 54.17%, 26-50%: 25%, <25%: 8.33%  Reduction in pain: 66.67%  Reduction in itching: 79.17% | F: 1 year or until recurrence  R: 8 of 22 (36.36%) <6 months of last treatment | Hyperpigmentation: 12.5%  Pain at injection site: 4.17% |
| Sadeghinia 2012 [19], Iran | Number of patients and keloids NS (±20) | Face and neck, trunk, upper- and lower limbs | At 44-week follow-up (baseline)  Mean height (mm): approx. 1.50 (approx. 4.75)  Mean surface (mm^2^): approx. 1000 (approx. 1750)  Mean erythema (5-point scale): approx. 0.4 (approx. 4.4)  Mean induration (5-point scale): approx. 2.5 (approx. 4.4)  Mean pruritus (5-point scale): approx. 3.5 (approx. 4.4.)  Patient self-assessment: good-excellent: 40%, poor-fair: 60%  Observer assessment: good-excellent: 50%, poor-fair: 50% | F: 32 weeks  R: NS | None |
| Prabhu 2012 [23], India | 15 patients with 15 keloids | Shoulder (7), upper limb (1), chest (6), back (1) | Volume, change: 71.23% ± 18.01  VAS pain, change: 24.00% ± 17.45  Consistency: NS  Clinical appearance (atrophic, hypertrophic, nodular): NS | F: 6 months  R: None | Increased pruritus: 6.7% |
| Salem 2009 [29], Egypt | 10 patients with 10 keloids | Neck, face, breast, forearm, earlobe, mastoid region, deltoid region | Degree of flattening and size reduction: >75%: 50%, 51-75%: 30%, 25-50%: 10%, <25%: 10%  VEGF expression (brown deposits in fibroblasts and endothelial cells), staining: weak: 60% (control: 70%, baseline: 10%); moderate: 30% (control: 20%, baseline: 0%); strong: 10% (control: 10%, baseline: 80%) | F: 1 year  R: None | Pain: 40%  Hypertrichosis: 20% |
| Anchlia 2009 [20], India | 11 patients, number of keloids NS | NS | Volume; at one month post-treatment and maintained in the 2^nd^ and 3^rd^ month: maximum response (objective flattening): 100%, minimum improvement: 20%.  Response, average: 43.98%  Overall response to therapy: 1-25%: 27.27%, 26-50%: 63.64%, 51-100%: 9.09% | F: 3 months  R: NS | NS |
| Koc 2008 [14], Turkey | 9 patients with 9 keloids | Head or neck (2), upper extremity (4), thorax (7) | NS for keloids | F: 2 months  R: NS | None |
| Berman 2008 [27], United States | 9 patients, number of keloids NS | NS | Median percent change at week 8 from baseline:  Lesion size: length: -5, height: -32, width: -17, volume: -68  Lesion assessment: induration: -44, erythema: -44, pigmentary alteration: 92, pain: -24, pruritus: -23  Cosmetic assessment: investigator: -40, patient: -42  Patient satisfaction scale: 62 | F: 4 weeks  R: NS | None |
| Layton 1994 [39], United Kingdom | 11 patients, number of keloids NS | Face, back, chest | Degree of response:  Face: 0%: 50%, 1-25%: 50%  Chest: 1-25%: 50%, 26-50%: 50%  Back: 0%: 10%, 1-25%: 10%, 26-50%: 45%, 51-100%: 30%  Lesions of <6 mm depth on the back flattened more than those on the chest (p < 0.03)  Grossly palpable keloids >6mm in depth: no response  Moderately palpable lesions: better response than flatter lesions  Keloids with 1.5-2 times higher bloodflow: better response than less vascular lesions | F: 8 weeks  R: NS | Discomfort at the time of administration |
| Usanakornkul 2017 [21], Thailand | 40 patients with 40 keloids | Ear lobule (10), shoulders (18), sternum (18) | Mean VAS pain:  TAC: needle-puncture 4.18 ± 2.12, intralesional injection 4.97 ± 2.50  TAC + 1% lidocaine in 1:1: needle-puncture 3.82 ± 2.48 (p = 0.488), during injection 4.97 ± 2.79 (not significant)  EMLA 1 hour before TAC: needle-puncture 2.03 ± 2.02 (p < 0.001), during injection 4.10 ± 2.80 (not significant)  EMLA 1 hour before TAC + 1% lidocaine in 1:1: needle-puncture 2.20 ± 1.99 (p < 0.001), during injection 4.43 ± 2.68 (not significant)  Pain-relief duration and methods of injection: no significant difference | NA | NS |
| Wang 2017 [15], China | 17 patients with 21 keloids | Chest (9), shoulder (3), back (1), arm (3), mandible (1) | Mean VAS pain, p < 0.01: without cryotip: 8.2, with cryotip: 2.5  Verbal descriptor scale, p <0.01:  Without cryotip: moderate (12%), severe (18%), extreme (71%)  With cryotip: no pain (5.9%), mild (82%), moderate (12%) | NA | NS |
| Jongkajornpong 2021 [22], Thailand | 17 patients with 21 keloids | Ear (10), neck (1), back (2), chest (10), abdomen (5), upper extremities (11), lower extremities (4) | Mean VAS pain:  Control: needle prick 4.26, infiltration 5.34, 1 hour after injection 2.60  Skin cooling: needle prick 1.09 (p < 0.001), infiltration 1.78 (p < 0.001), 1 hour after injection 1.84 (not significant)  EMLA: needle prick 2.34 (p < 0.05), infiltration 3.39 (p < 0.05), 1 hour after injection 2.29 (not significant)  Patient satisfaction: control 3.07, skin cooling 4.41 (p < 0.001 compared with control and EMLA), EMLA 3.36 (not significant compared with control) | NA | None for ice packing |

Supplement 3 presents the summarized treatment results. Moreover, study design related data are presented, including outcome measures, follow-up period, recurrence and adverse events.

NS: not specified; NA: not applicable; VSS: Vancouver Scar Scale, POSAS: Patient and Observer Scar Assessment Scale; VRS: verbal rating scale; IQR: interquartile range; SEM: standard error of the mean

*: Data difficult to acquire from graph or diagram
